# Quantification of Anisotropy in Exchange and Dispersion Interactions: A Simple Model for Physics-Based Force Fields

**DOI:** 10.1021/acs.jpclett.4c02034

**Published:** 2024-09-24

**Authors:** Kristian Kříž, David van der Spoel

**Affiliations:** Science for Life Laboratory, Department of Cell and Molecular Biology, Uppsala University, Husargatan 3, Box 596, SE-75124 Uppsala, Sweden

## Abstract

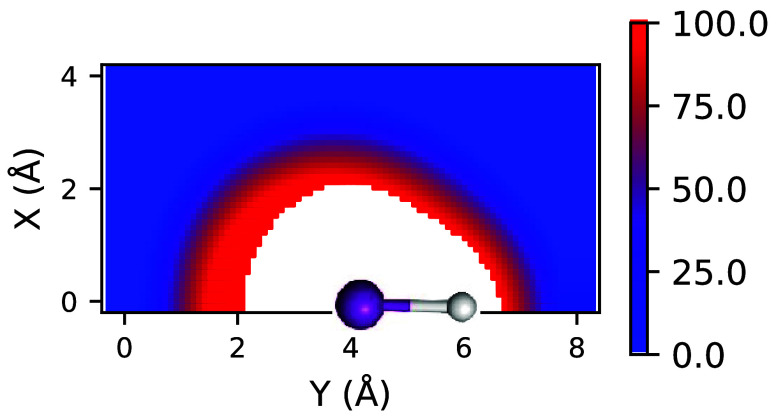

In some compounds, exchange repulsion is orientation dependent. However, in contrast to quantum chemical methods that treat exchange explicitly, empirical models assume exchange to be spherically symmetric, yielding an average description only. Here we quantify the anisotropy of exchange and dispersion energy for hydrogen halides and water by probing these compounds with a helium atom using the symmetry-adapted perturbation theory (SAPT). The exchange interaction is reduced by up to 33% due to the σ-hole in hydrogen iodide, depending on the location of the probe. We demonstrate how this anisotropy can be modeled in empirical force fields either using an angle-dependent potential or by introducing virtual sites, reducing the error in the empirical model by a factor of 5 compared to isotropic atoms. Lone-pairs on water, positioned close to perpendicular to the plane of the molecule, on a line with the oxygen atom, and, surprisingly, σ-holes on water both modulate the exchange interaction strongly. Both lone-pairs and σ-holes can be modeled by virtual sites, leading to an 80% reduced error.

Symmetry adapted perturbation theory (SAPT) is a powerful computational chemistry method that provides a means of decomposition of the interaction energy into physically meaningful components.^1^ For this reason, SAPT calculations have been proposed to provide reference data for fitting of parameters of force fields,^2^ and data sets have been published to further this goal;^3^ ref (4) provides a review of quantum chemistry data sets. Training force fields to reproduce energy components rather than total interaction energies reduces the space for error-cancellation and thus is expected to produce more physical and transferable models.^5^ Additionally, the use of “independent” energy components to which empirical functions can be fitted one by one reduces the complexity of the interaction-energy-parameter hyperspace, facilitating systematic design of force fields.^6^ In most force fields, it is tacitly assumed that atoms are spherical, at least with regard to exchange and dispersion interactions. Here, we utilize the analytical power of SAPT calculations to probe the geometry dependence of the energy components and evaluate whether this assumption is valid.

A σ-hole is an electron-depleted volume on a bonded atom located on the side of the atom opposite to the covalent bond(s) that the atom is forming. The participation of p electrons in a σ bond leaves the nonbonding lobe electron deficient (σ-hole), as the bonding electron is now localized in the formed bond. The magnitude of σ-holes can vary depending on the electronegativity of substituent atoms.^7^ This particular form of anisotropy on halogen atoms results in the potential to form halogen bonds, which were the first described σ-hole interactions.^7^ Halogen bonds^8^ have characteristics such as tunability,^9−11^ which make them interesting for studies in drug design and related fields.^12^

Some force fields account for the presence of σ-holes by introducing a positively charged virtual site at the σ-hole position.^16,17^ In this manner, anisotropy in electrostatics is addressed. However, the electrons are responsible for more interactions than just those that can be modeled by the Coulomb law. The anisotropy around halogen atoms was addressed by a specific force-field employing a cosine-dependent function, scaling both Coulomb and exchange terms.^18,19^ The model presented by those authors was judged to be cumbersome^20^ and hence has not received large acceptance by the community yet. Further models were derived by Ponder and co-workers based on multipole expansion^21,22^ or a p-orbital to include directionality.^23^ In this work, we use simpler approaches that are applicable immediately to the vast majority of force fields, while we quantify the expected gain of precision in terms of root-mean-square error (RMSE).

Anisotropy is considered in the crystal structure prediction community as well, as highlighted, for example, by a recent paper by Aina et al. on trinitrobenzene.^24^ Indeed, modeling organic crystals by force fields is far from straightforward and isotropic nonpolarizable force fields may not suffice for this purpose.^25,26^ The work by Aina and other recent models^27^ derive to some extent from a visionary paper by Stone and Price dating back to 1988,^28^ showing that model adoption lags model development by decades. Common to all these models is that they require considerable additional computational effort, which is why a simpler model that covers most of the anisotropy in dispersion and exchange interactions may yet be worthwhile.

The extent of anisotropy in the exchange, dispersion, electrostatics, and induction interactions as derived from SAPT calculations is illustrated here using angular scans of hydrogen halides or water interacting with a helium probe. Scans of water probed with a hydrogen fluoride molecule are available in the SI but are not discussed here, as they are very similar to the He-probing for exchange and dispersion. This analysis allows us to “discover” the important players, σ-holes and lone pairs, that is, their approximate location and magnitude, and to devise strategies to incorporate the anisotropies caused by them into empirical models. For exchange and dispersion, we fit a not-corrected “Spherical” Buckingham potential,^29^ as well as Buckingham potentials corrected using either a cosine term or a virtual site to SAPT energies on a dense regular grid of helium probes placed around the model systems (Figure S1). In contrast to the earlier model^18−20^ that was fitted to the total interaction energy, this approach does not rely on compensation of errors between energy terms and therefore is expected to yield force field parameters that are transferable. In addition, the Lennard-Jones potential used in that previous work^18−20^ is not sufficiently flexible to accurately model exchange and dispersion.^30^

For hydrogen iodide interacting with a helium probe, the exchange energy resembles a cosine-like function of the angle, and this angular dependence of exchange is preserved up to large separations (Figure 1A). For water, two orientations were used, one with a probe atom “moving” in a plane containing all water atoms (frontal plane, Figure 1B) and a plane that reflects the hydrogen atoms on each other (sagittal plane, Figure 1C). Interestingly, for the frontal water model interacting with helium, the anisotropy in SAPT components around the oxygen strongly suggests the presence of σ-holes (Figures 1B, S2–S6). For instance, the exchange interaction displays a minimum in the region corresponding approximately to an extension of the covalent H–O bond (Figure 1B). This is noteworthy since σ-holes on oxygen compounds are usually not deemed significant.^31^ On the other hand, the reduction of dispersion due to the σ-hole is small (Figure S2), likely because it is compensated for by the strengthening of dispersion by the adjacent hydrogen atoms as the probe gets closer to them. For the sagittal water model, Figure 1C shows that the potential rises with no clearly apparent maximum corresponding to lone pairs, up to an angle where the probe interaction with adjacent hydrogen atoms is dominating. Angular scans of other model systems and other SAPT components can be found in Figures S2–S31.

**Figure 1:**
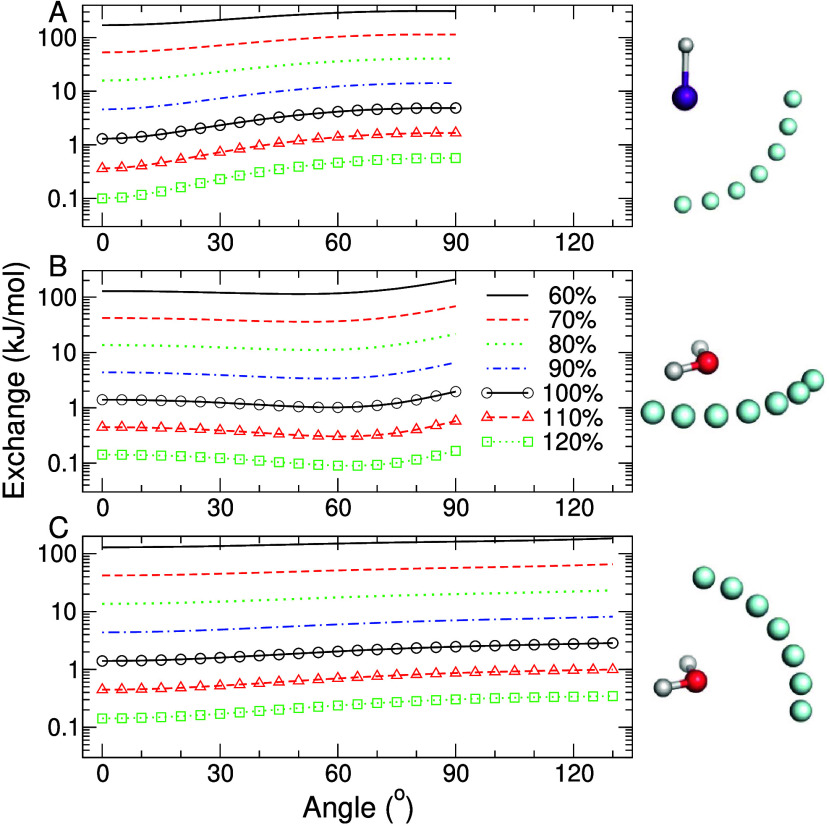
SAPT exchange (A) of hydrogen iodide as a function of angle H–I–He at distances corresponding to percentages of the sum of the van der Waals radii of iodine (1.98 Å) and the helium probe (1.4 Å), (B) for the frontal plane water model, and (C) for the sagittal plane water model, both as a function of angle and distance from the oxygen atom (van der Waals radius 1.52 Å) in the same manner as for iodine. Angle for panel B is that between the water bisector and the O–He vector in the plane of the molecule and for panel C is that perpendicular to the plane of the molecules (Figure S1). The SAPT2+(CCD)δMP2 method^13^ was used with an augmented triple-ζ basis set,^14^ and calculations were performed using the Psi4 software suite.^15^

Parameters of empirical van der Waals dispersion and exchange potentials were fitted to 2D energy component grids of size 4 × 8 Å^2^ with a 0.1 Å spacing. The grid points were computed as described in the caption of Figure 1. For the frontal and sagittal planes of water, the grids were 3 × 3 Å^2^ and 3 × 3 × 3 Å^3^, respectively (Figure S1). The halogen was placed on the [0, 4] Å point, with hydrogen placed above the halogen, at the respective experimental covalent distance. Similarly, in the case of water models, oxygen was placed in the center of the grid, at [3, 3(, 3)] Å. A helium atom was placed in every grid point that was further away than half the sum of the van der Waals radii of either hydrogen halide or water elements. Remaining overly repulsive separations were filtered out later, based on an exchange energy threshold of 100 kJ/mol. For hydrogen halides from F to I this threshold value corresponds approximately to orientations with helium being positioned between 60% and 70% of the sum of the van der Waals radii of halogen and helium. Although 60% is arguably very close, separations comparable to these occur, for instance, in the hydrogen bonded hydrogen-fluoride dimer, and thus short distance are highly relevant. In addition to helium, neon was used with hydrogen iodide to verify the independence of the results on the noble gas used (Tables S13, S14).

In addition to a standard “Spherical” Buckingham potential,^29^ we introduce a “Cos” Buckingham potential modulated by a cosine function scaled by parameters *W* for exchange or *Q* for dispersion, respectively. The angle prefactor *k* was limited to *k* ≤ 2 in order not to introduce unwanted periodic behavior. For the hydrogen halides, the relations were
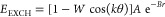
1for exchange and, analogously, for the dispersion:
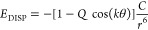
2where the θ is the angle between the hydrogen, the halogen, and the probe for hydrogen halides. For hydrogen halides, the maximum angle is 90°, after which it is set to a constant 90° to prevent a modulation of the interaction due to spurious periodicity in regions where there is no σ-hole. The addition of an angular term on the halogen (eq 1) decreased the root-mean-square error (RMSE) of the fit to the SAPT exchange energy by a factor of 5 (Table S1). This improvement seemed insensitive to the variation of the exchange cutoff threshold, as the RMSE reductions using a 50 kJ/mol exchange threshold instead of 100 are also at least a factor of 5 (Table S15). In the Cos X model, the parameter *W*_*x*_ for iodine is 0.34, which means the exchange energy is reduced by 34% going from 0 to 90°. This is a large change in energy that is likely to contribute significantly to relative orientations of interacting molecules. Introducing an additional angular term on the hydrogen makes virtually no difference (Table S1). Indeed, the exchange due to the s-orbital of the bound hydrogen atoms can with good accuracy be approximated by just a spherically symmetric potential. However, and notably, for hydrogen fluoride, there was a moderate improvement of the RMSE for the angularly augmented exchange potential (eq 1) on hydrogen atoms (Table S7). It is likely the case that the strong polarization due to the bond to the highly electronegative fluorine exerts a pull on the hydrogen electron, forming an anisotropy that is most pronounced on the hydrogen apex, similarly as in the case of a σ-hole.

Seeing the impact of the angular dependence on the exchange interaction potential for hydrogen halides, we explored whether a virtual site is able to cover the anisotropy. A site was added on the apex of the halogen, interacting with the probe atom by a simple exponential term:
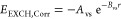
3The attractive term is thus designed to dampen the exchange that is overly repulsive at the σ-hole site. The position of the virtual site was varied to achieve the best fit. Table S1 shows that a virtual site at 0.9 Å from the iodine in practice yields the same RMSE as the more cumbersome angle-dependent potential. Seeing that virtual sites are used already in force fields to model the electrostatic effect of σ-holes,^16,17^ adding this simple correction (eq 3) to the exchange may be preferable. However, the optimal distances of these exchange-specific virtual sites to their halogen atoms are smaller than the ones of the virtual sites used for electrostatics.^16,17^Table 1 summarizes the extent of the anisotropy observed in the exchange interactions after fitting to empirical functions. Not surprisingly, the σ-hole is stronger in the heavy halogens than in the lighter ones.

**Table 1:** Summary of Anisotropy (*W*) from Fitting SAPT Exchange Using Eq 1 for Hydrogen Halides and Reduction δ of Fitting RMSE by Introducing New Models Based on Those Equations or Using a Virtual Site

compound	HI	HBr	HCl	HF
*W* (%)	34	31	28	19
δ Cos (%)	80	83	84	77
δ Vsite (%)	81	84	85	86

Introduction of an angular term for dispersion (eq 2) reduces the RMSE from fitting the SAPT energy by almost a factor of 3 for hydrogen iodide, and the parameter *Q*_*x*_ is 0.2, corresponding to 20% difference in dispersion energy between the 0 and 90° probe positions (Table S2). For the other hydrogen halides, the reduction is less significant, though (Tables S4, S6, and S8).

For the water models, the exchange is modified according to
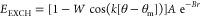
4where θ_m_ is the displacement of the angle of the minimum or maximum in the energy, optimized as a free parameter. θ_m_ is expected to indicate the position of σ-holes and lone pairs, respectively. In the case of water models with the probe atom moving in the molecular plane (frontal plane), the angular term was negative, subtracting exchange on a σ-hole, while for the sagittal model covering lone pairs, the angular term was positive instead corresponding to a negative value for *W*.

A 3-fold reduced RMSD was found for the exchange of frontal water (Table S9) and for the sagittal water model RMSE was reduced by a factor of 5 (Table S11) upon implementing eq 4. For both water models, the *k* parameter is closer to 1 compared to hydrogen halides, where it tends to be closer to 2. The fit of eq 4 to the SAPT exchange allowed estimation of the location of the σ-holes and lone pairs in water. Keeping in mind that a rather simple empirical model is used here, the σ-holes due to exchange were found to be located ≈65° from the oxygen apex (Table S9), bending toward hydrogen atoms, rather than being straight in the extension of the covalent bond. This displacement toward hydrogen atoms obtained from the cosine term fitting was slightly larger than what would one expect based on results of angular scans (Figure 1). There, one needs to remember that the minimum of the potential is due to interaction with both oxygen (σ-holes) and adjacent hydrogen atoms, while empirical fit allows these two to be separated. Interestingly, the lone pairs determined by the sagittal scan are also much further from the expected tetrahedral orientation, close to 95° away from the oxygen apex (Table S11). This finding is consistent with earlier research;^32^ however it contradicts the still popular notion of tetrahedrally positioned lone pairs on water, even though some force fields have already adopted the perpendicular lone-pair position.^33^Figure 2 illustrates the positions of the σ-holes and lone pairs on water as derived from the fits (Tables S9 and S11). The frontal and sagittal scans of the exchange around water were then combined in a fit of a single model with no virtual sites or virtual sites on lone-pairs, on σ-holes, or both. A residual correlation plot is given in Figure 3. Interestingly, adding virtual sites on the σ-holes reduces the RMSE more in the fit to all the data than adding virtual sites on the lone pairs. Adding virtual sites on both σ-holes and lone-pairs decreases the RMSE even further. The positions of the virtual site were part of the fit.

**Figure 2:**
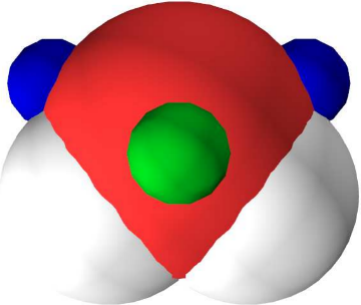
Positions of σ-holes (blue) and lone pairs (green) in water determined by fitting the exchange interaction.

**Figure 3:**
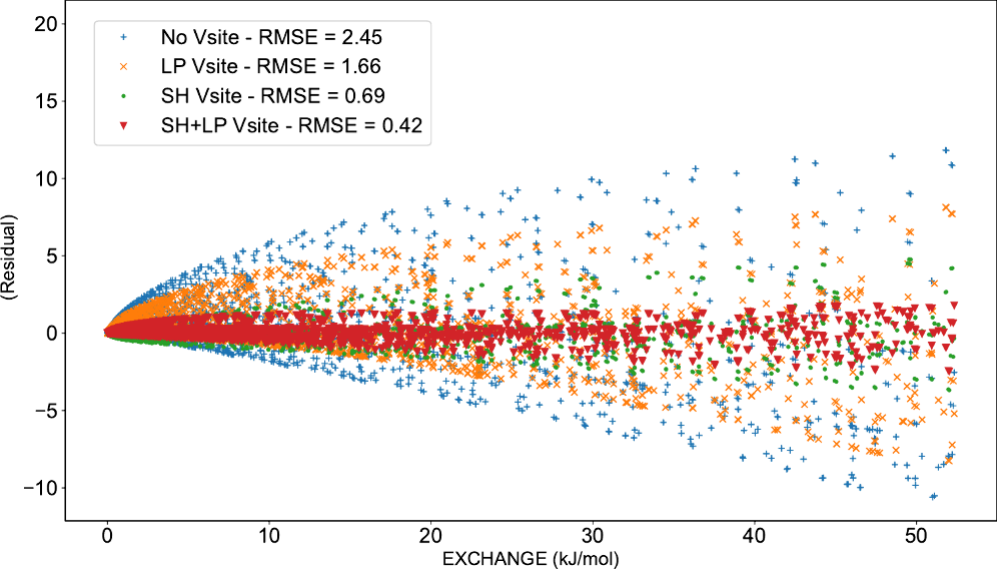
Residual plot comparing the exchange energies from the SAPT calculations (*x*-axis) to the fitted one with no virtual sites or virtual sites on the lone-pairs (LPs), the σ-holes (SH), or both. The root-mean-square error is indicated.

The modulated energy equation for dispersion for water used is
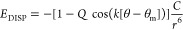
5Tables S10 and S12 show that the reduction in RMSE when fitting eq 5 to the SAPT dispersion is less than a factor of 2. In absolute numbers, the RMSE are considerably smaller than those for exchange, and in both cases, the prefactor *k* and the displacement angle θ_m_ differ from the corresponding values obtained in fitting the SAPT exchange energy (Tables S9 and S11 respectively). In light of these observations, it may be sufficient to continue to treat dispersion spherically in force fields.

The modifications suggested here present a small to moderate tax on computational efficiency. Most hydrogen halide models include a virtual site for Coulomb interactions already^34,35^ and adding an exchange correction (or simply van der Waals interactions) should require a minor increase in computer time only. Regarding water, in a nonpolarizable model with 3 atoms only, adding virtual sites on the lone-pairs (as is also done in water models like TIP5P^36^), increases the calculation time roughly by the square of 5/3. Adding virtual sites on the σ-holes in water would increase the computer time further. Although it is possible to implement cosine-dependent potentials in, for instance, OpenMM,^37^ it is difficult to estimate the effect on total computer time.

We suggest that properly addressing the exchange anisotropy is necessary in the development of accurate physics based force fields, not only with regard to a better description of halogen bonds but likely in general as well. Indeed, the reduction in RMSE is well over 70% in most cases (Table 1, Figure 3). These findings are highly relevant since accurate models of halogen bonding are important, for instance, in drug design^38,39^ or in membrane biology.^40^ Implementation of three-particle based nonbonded potentials for halogenic compounds was deemed impractical,^20^ but our analysis shows (Table 1) that even more accurate exchange energies can be obtained by introducing an exchange correction on a virtual site close to the halogen particle. Although more elaborate models of anisotropy in small molecules have been proposed,^21−24,27,41^ the here presented simple model based on virtual sites may be easier to incorporate in standard biomolecular force fields that already implement virtual sites to model the electrostatic anisotropy due to σ-holes.^16,17^

## Supporting Information Available

Additional tables with force field parameters and figures with energy scans (PDF)
Transparent Peer Review report available (PDF)
