# Supplementary material for: Quantification of Anisotropy in Exchange and Dispersion Interactions: A Simple Model for Physics-Based Force Fields
Source: J Phys Chem Lett. 2024 Sep 24;15(39):9974–8. doi: 10.1021/acs.jpclett.4c02034 (PMC11457221; doi:10.1021/acs.jpclett.4c02034)
Supplement: Supplementary file 1 — jz4c02034_si_001.pdf [file jz4c02034_si_001.pdf]

Quantification of Anisotropy in Exchange and Dispersion Interactions: A Simple Model for Physics-Based Force Fields - Supporting Information

Kristian Kříž and David van der Spoel*

Science for Life Laboratory, Department of Cell and Molecular Biology, Uppsala University, Husargatan 3, Box 596, SE-75124 Uppsala, Sweden

E-mail: david.vanderspoel@icm.uu.se

Table S1: Parameters A (kJ/mol), B (1/Å), W, k and corresponding RMSE of different models for exponential exchange repulsion for hydrogen iodide. The Vsite parameter denotes the distance of the virtual site to the halogen .

Model	A_x	B_x	W_x	k_x	A_x	B_h	W_h	k_h	RMSE
Spherical	46694	2.70	N/A	N/A	5920	3.21	N/A	N/A	4.94
Cos. X	160055	3.18	0.34	2.00	13423	3.79	N/A	N/A	0.98
Cos. X, H	159088	3.18	0.33	2.00	16003	3.88	0.07	2.00	0.95
Model	A_x	B_x	A_{vs}	B_{vs}	A_h	B_h	$Vsite_{dist}$ (Å)	RMSE	
Vsite X	146506	3.03	51.33	2.59	15097	3.88	0.90	0.94	

Table S2: Parameters C (kJ/mol Å^6), Q, k and corresponding RMSE of different models for sixth-power dispersion attraction for hydrogen iodide.

Model	C_x	Q_x	k_x	C_h	RMSE
Spherical	2321	N/A	N/A	90	0.61
Cos. X	2312	0.20	2.00	78	0.23

Table S3: Parameters A (kJ/mol), B (1/Å), W, k and corresponding RMSE of different models for exponential exchange repulsion. Hydrogen bromide. The Vsite parameter denotes the distance of the virtual site to the halogen.

Model	A_x	B_x	W_x	k_x	A_x	B_h	W_h	k_h	RMSE
Spherical	66895	3.03	N/A	N/A	5039	3.23	N/A	N/A	4.03
Cos. X	171140	3.43	0.31	2.00	10115	3.76	N/A	N/A	0.68
Cos. X, H	169100	3.43	0.31	2.00	13075	3.90	0.09	2.00	0.60
Model	A_x	B_x	A_{vs}	B_{vs}	A_h	B_h	$Vsite_{dist}$ (Å)	RMSE	
Vsite X	170307	3.30	68.42	2.92	11727	3.88	0.70	0.65	

Table S4: Parameters C (kJ/mol Å^6), Q, k and corresponding RMSE of different models for sixth-power dispersion attraction. Hydrogen bromide.

Model	C_x	Q_x	k_x	C_h	Q_h	k_h	RMSE
Spherical	1561	N/A	N/A	67	N/A	N/A	0.51
Cos. X	1564	0.19	1.79	58	N/A	N/A	0.23

Table S5: Parameters A (kJ/mol), B (1/Å), W, k and corresponding RMSE of different models for exponential exchange repulsion. Hydrogen chloride. The Vsite parameter denotes the distance of the virtual site to the halogen.

Model	A_x	B_x	W_x	k_x	A_x	B_h	W_h	k_h	RMSE
Spherical	76848	3.26	N/A	N/A	4994	3.31	N/A	N/A	3.36
Cos. X	165656	3.60	0.28	2.00	9711	3.84	N/A	N/A	0.53
Cos. X, H	163192	3.60	0.27	2.00	11925	3.96	0.09	2.00	0.43
Model	A_x	B_x	A_{vs}	B_{vs}	A_h	B_h	$Vsite_{dist}$ (Å)	RMSE	
Vsite X	155696	3.45	55.74	2.95	11414	3.98	0.70	0.50	

Table S6: Parameters C (kJ/mol Å^6), Q, k and corresponding RMSE of different models for sixth-power dispersion attraction. Hydrogen chloride.

Model	C_x	Q_x	k_x	C_h	Q_h	k_h	RMSE
Spherical	1128	N/A	N/A	56	N/A	N/A	0.43
Cos. X	1138	0.18	1.69	49	N/A	N/A	0.22

Table S7: Parameters A (kJ/mol), B (1/Å), W, k and corresponding RMSE of different models for exponential exchange repulsion. Hydrogen fluoride. The Vsite parameter denotes the distance of the virtual site to the halogen.

Model	A_x	B_x	W_x	k_x	A_x	B_h	W_h	k_h	RMSE
Spherical	93314	3.98	N/A	N/A	3880	3.44	N/A	N/A	1.75
Cos. X	127905	4.13	0.19	1.63	6131	3.86	N/A	N/A	0.41
Cos. X, H	123686	4.13	0.16	1.90	11001	3.99	0.37	0.82	0.20
Model	A_x	B_x	A_{vs}	B_{vs}	A_h	B_h	$Vsite_{dist}$ (Å)	RMSE	
Vsite X	117125	3.94	78.01	3.41	7453	4.06	0.30	0.25	

Table S8: Parameters C (kJ/mol Å^6), Q, k and corresponding RMSE of different models for sixth-power dispersion attraction. Hydrogen fluoride.

Model	C_x	Q_x	k_x	C_h	Q_h	k_h	RMSE
Spherical	359	N/A	N/A	32	N/A	N/A	0.32
Cos. X	74929	1.00	0.03	28	N/A	N/A	0.23

Table S9: Parameters A (kJ/mol), B (1/Å), W, k, θ_m (°) and corresponding RMSE of different models for exponential exchange repulsion. Water, in plane passing all atoms (frontal).

Model	A_o	B_o	W_o	k_o	$\theta_{m,o}$	A_h	B_h	W_h	k_h	RMSE
Spherical	114568	3.96	N/A	N/A	N/A	6047	3.59	N/A	N/A	2.87
Cos. O	153774	3.94	0.35	1.17	65.32	8334	3.89	N/A	N/A	0.82
Cos. O, H	153755	3.94	0.35	1.17	65.3	153444	3.89	0.95	0.00	0.82

Table S10: Parameters C (kJ/mol Å^6), Q, θ_m(°), k and corresponding RMSE of different models for sixth-power distance dispersion attraction. Water, in plane passing all atoms (frontal).

Model	C_o	Q_o	$\theta_{m,o}$	k_o	C_h	RMSE
Spherical	494	N/A	N/A	N/A	41	0.61
Cos. O	683	0.33	38.4	1.63	22	0.35

Table S11: Parameters A (kJ/mol), B (1/Å), W, k, θ_m (°) and corresponding RMSE of different models for exponential exchange repulsion.Water a plane projecting hydrogen atoms on each other (sagittal)

Model	A_o	B_o	W_o	k_o	$\theta_{m,o}$	A_h	B_h	RMSE
Spherical	100459	3.76	N/A	N/A	N/A	3164	3.13	1.74
Cos. O	79421	3.70	-0.27	0.93	95.1	10129	3.78	0.32

Table S12: Parameters C (kJ/mol Å^6), Q, θ_m (°), k and corresponding RMSE of different models for sixth-power distance dispersion attraction. Water in plane between the hydrogen atoms (sagittal)

Model	C_o	Q_o	$\theta_{m,o}$	k_o	C_h	RMSE
Spherical	530	N/A	N/A	N/A	69	0.32
Cos. O	536	0.09	114.6	1.50	62	0.25

Table S13: Parameters C (kJ/mol Å$_6$), Q, k and corresponding RMSE of different models for sixth-power dispersion attraction for hyrdogen iodide. The probe atom used here was neon rather than helium.

Model	C_x	Q_x	k_x	C_h	Q_h	k_h	RMSE
Spherical	4465	N/A	N/A	224	N/A	N/A	0.51
Cos. X	4436	0.15	1.88	200	N/A	N/A	0.23

Table S14: Parameters A (kJ/mol), B (1/Å), W, k and corresponding RMSE of different models for exponential exchange repulsion. Hydrogen iodide. The Vsite parameter denotes the distance of the virtual site to the halogen. The probe atom used here was neon rather than helium.

Model	A_x	B_x	W_x	k_x	A_x	B_h	W_h	k_h	RMSE
Spherical	140053	2.84	N/A	N/A	13824	3.25	N/A	N/A	5.12
Cos. X	474259	3.28	0.33	2.00	33877	3.80	N/A	N/A	0.87
Cos. X, H	471047	3.28	0.33	2.00	43437	3.92	0.08	2.00	0.81
Model	A_x	B_x	A_{vs}	B_{vs}	A_h	B_h	$Vsite_{dist}$ (Å)	RMSE	
Vsite X	412151	3.13	74.96	2.66	38920	3.89	0.90	0.86	

Table S15: Parameters A (kJ/mol), B (1/Å), W, k and corresponding RMSE of different models for exponential exchange repulsion. Hydrogen iodide. The Vsite parameter denotes the distance of the virtual site to the halogen. Energy treshold 50 kJ/mol.

Model	A_x	B_x	W_x	k_x	A_x	B_h	W_h	k_h	RMSE
Spherical	33945	2.63	N/A	N/A	5813	3.24	N/A	N/A	2.91
Cos. X	177413	3.22	0.38	2.00	16474	3.90	N/A	N/A	0.51
Cos. X, H	177412	3.22	0.38	2.00	203940	3.90	0.92	0.00	0.51
Model	A_x	B_x	A_{vs}	B_{vs}	A_h	B_h	$Vsite_{dist}$ (Å)	RMSE	
Vsite X	155946	3.05	53.13	2.64	19306	4.01	0.90	0.51	

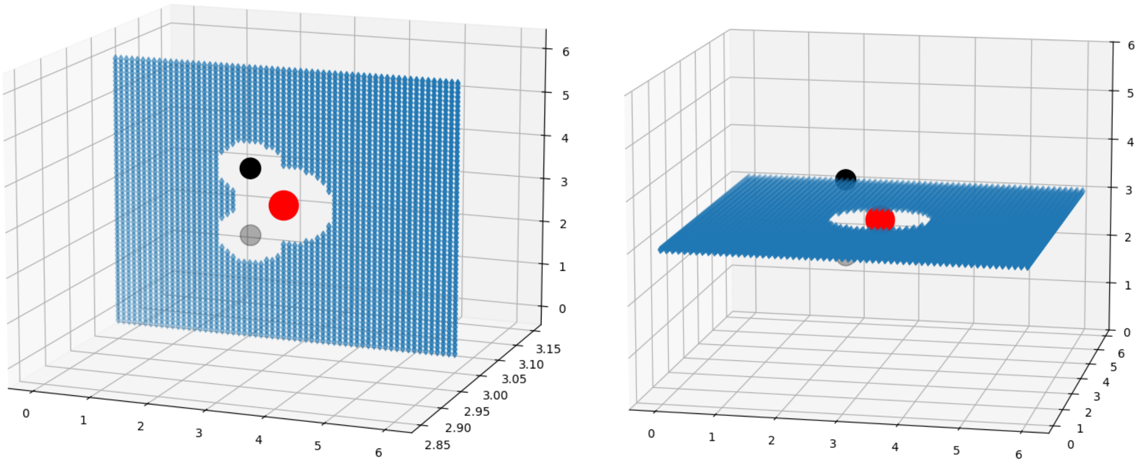

Figure S1: Grid points generated around water (frontal plane model left, sagittal right) used for the placing of the probe helium atom.

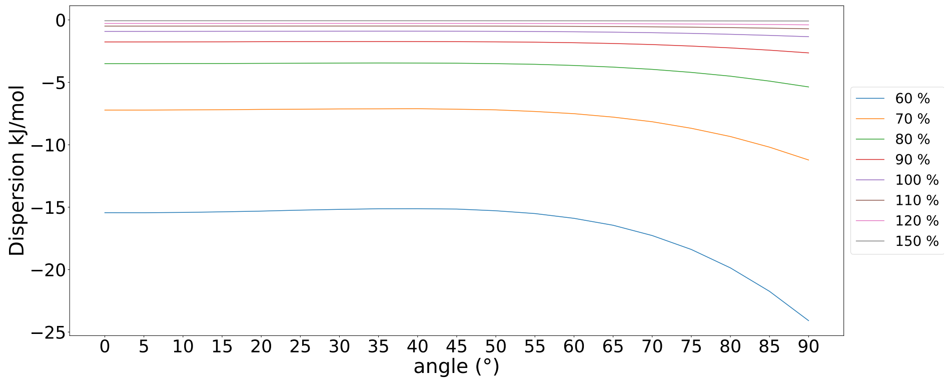

Figure S2: H2O-He, frontal dispersion

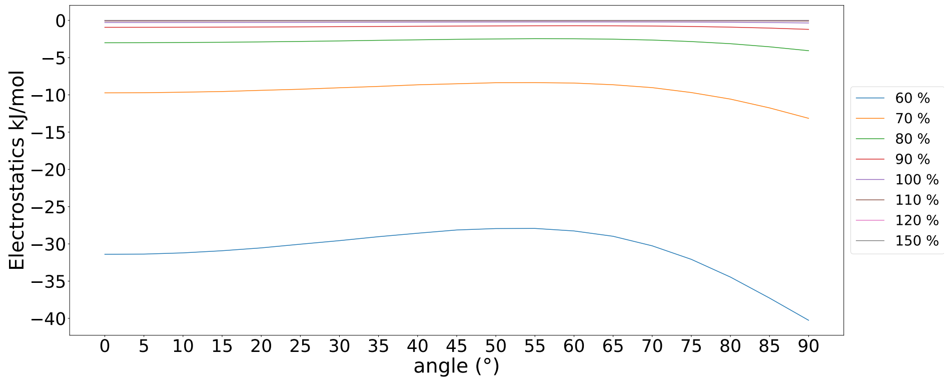

Figure S3: H2O-He, frontal electrostatics

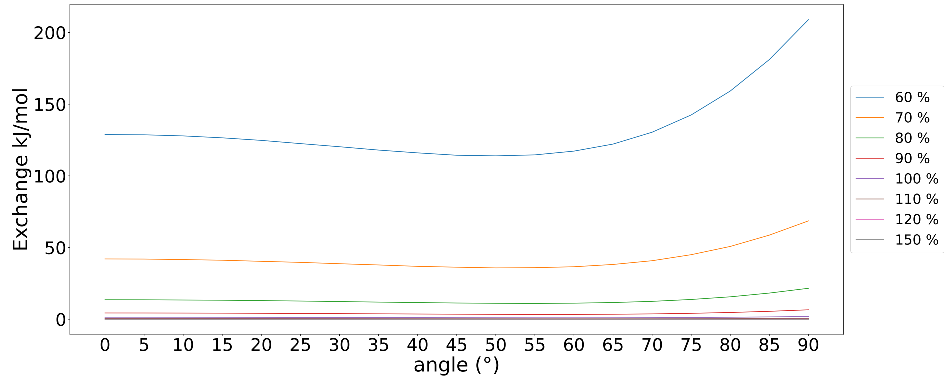

Figure S4: H2O-He, frontal exchange

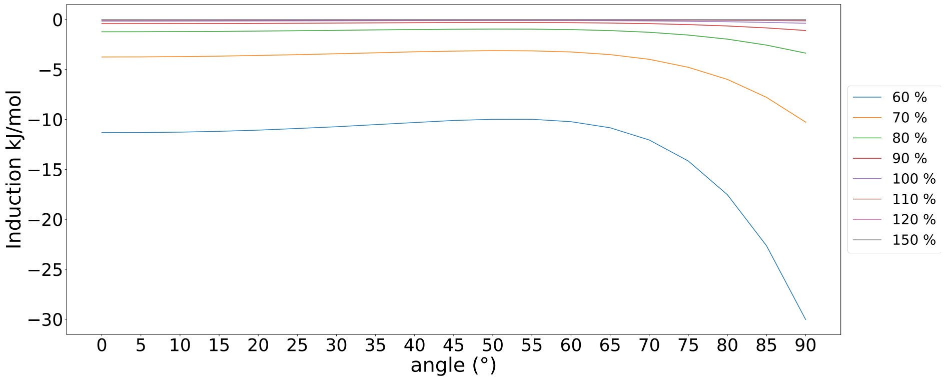

Figure S5: H2O-He, frontal induction

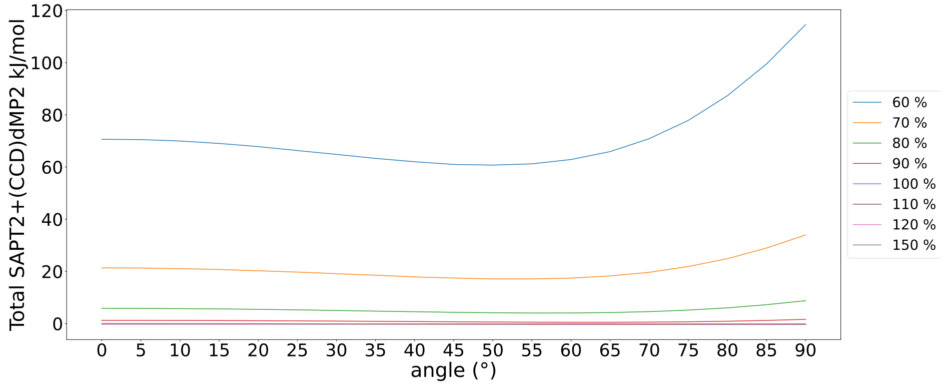

Figure S6: H2O-He, frontal total int,

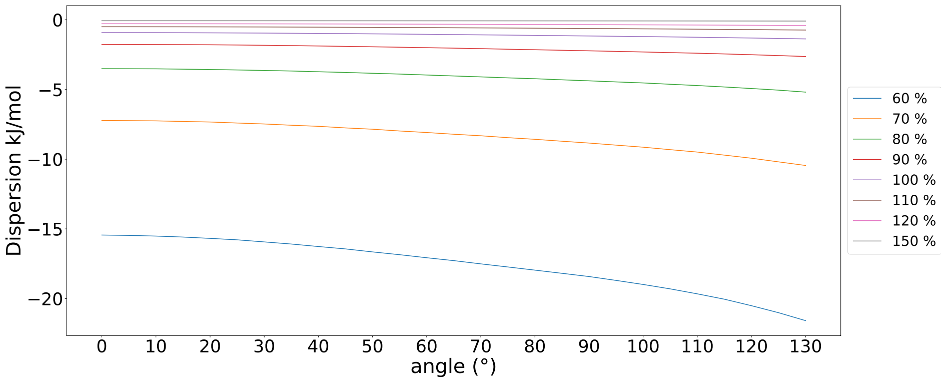

Figure S7: H2O-He, sagittal dispersion

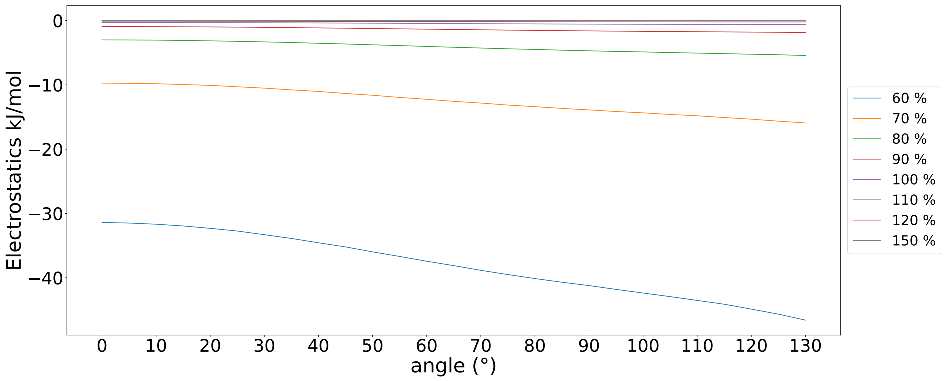

Figure S8: H2O-He, sagittal electrostatics

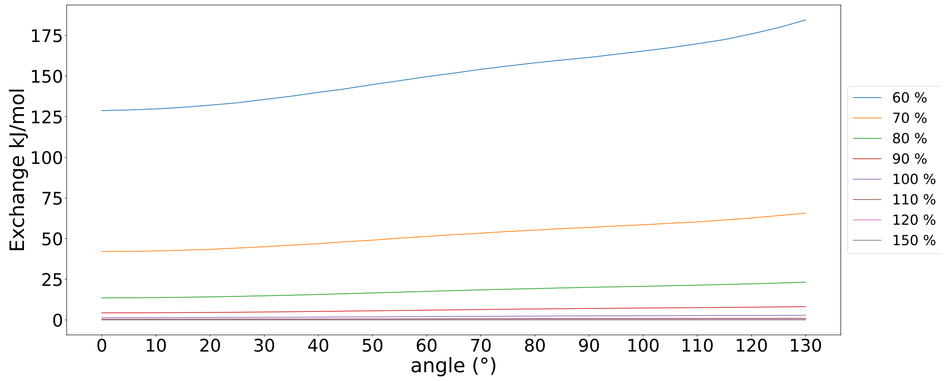

Figure S9: H2O-He, sagittal exchange

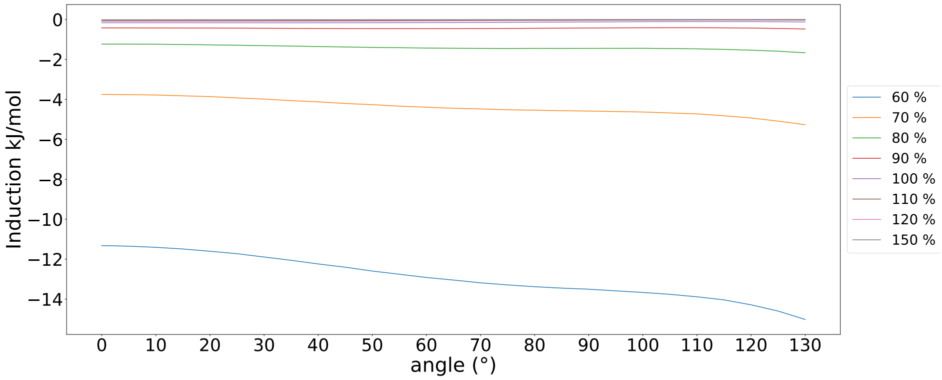

Figure S10: H2O-He, sagittal induction

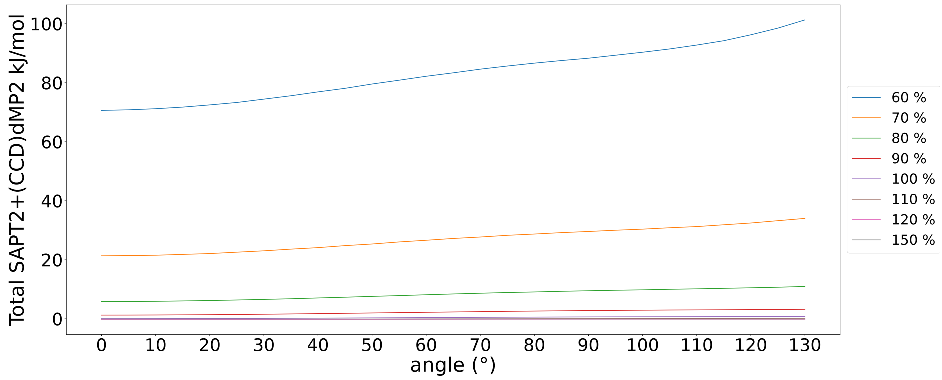

Figure S11: H2O-He, sagittal total int,

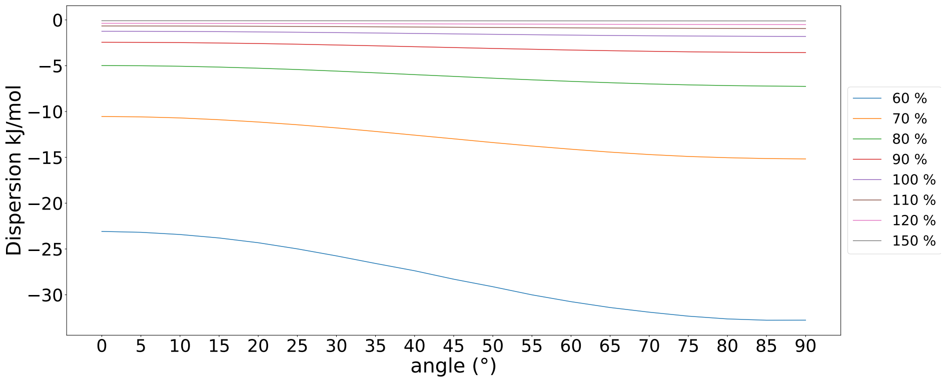

Figure S12: HI-He, dispersion

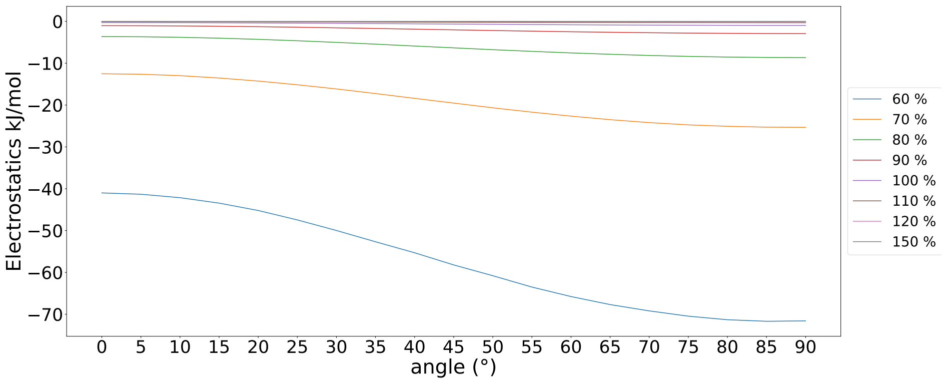

Figure S13: HI-He, electrostatics

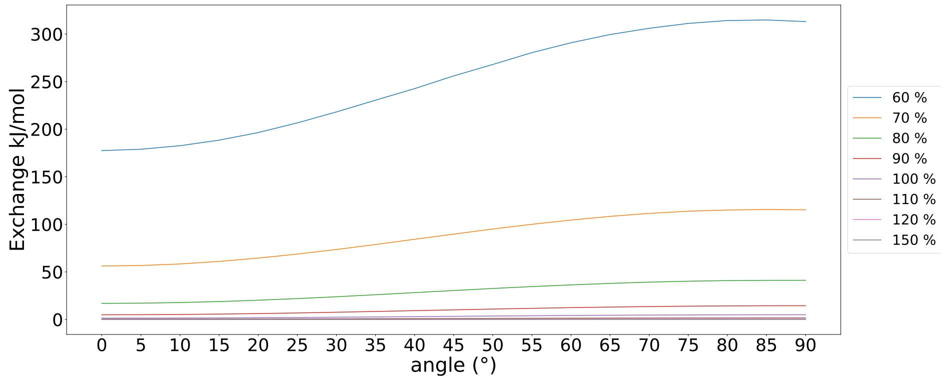

Figure S14: HI-He, exchange

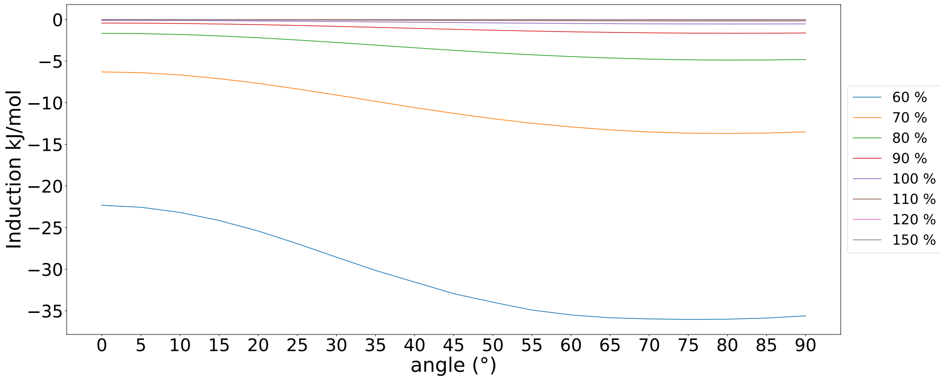

Figure S15: HI-He, induction

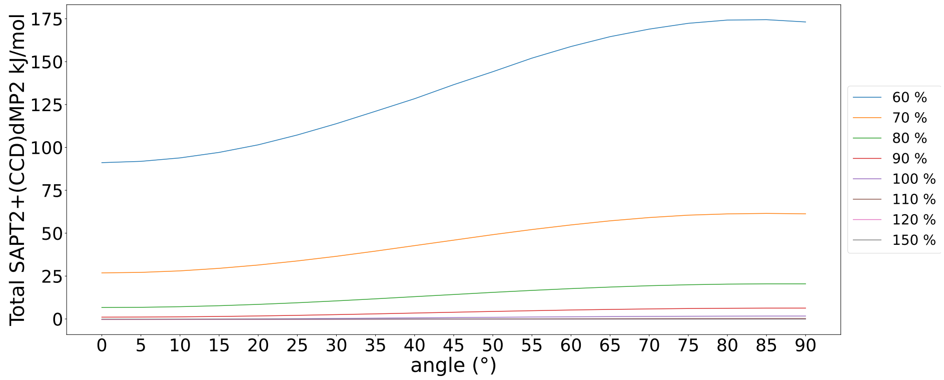

Figure S16: HI-He, total int,

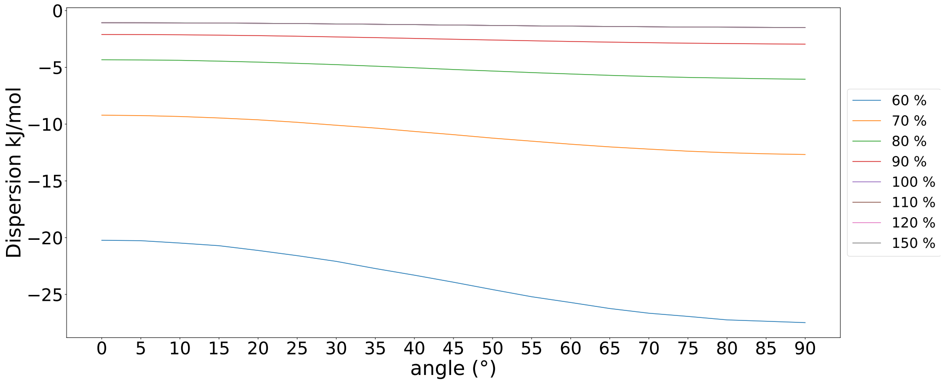

Figure S17: HI-Br, dispersion

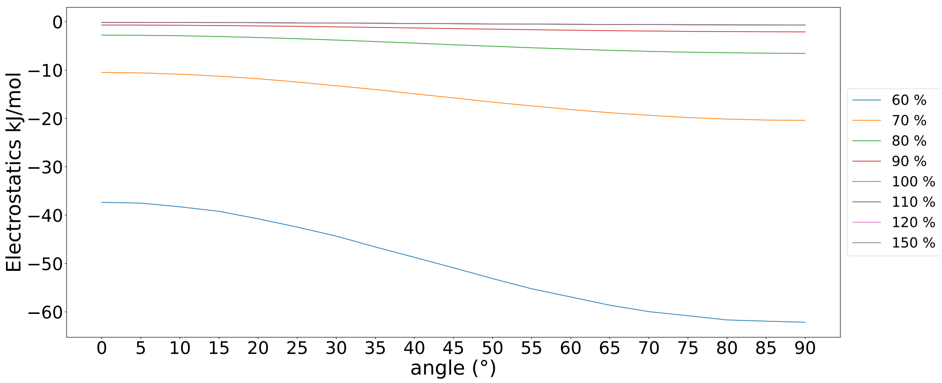

Figure S18: HBr-He, electrostatics

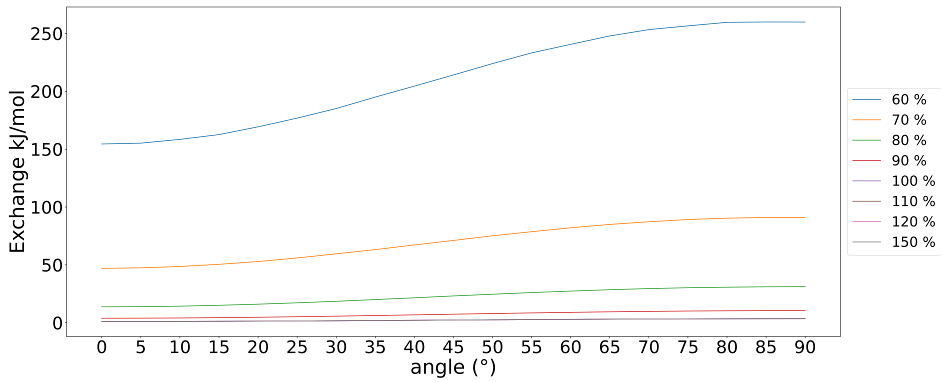

Figure S19: HBr-He, exchange

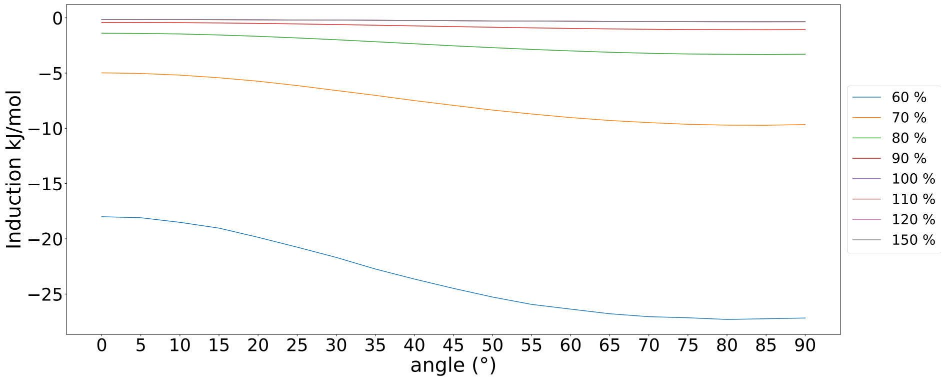

Figure S20: HBr-He, induction

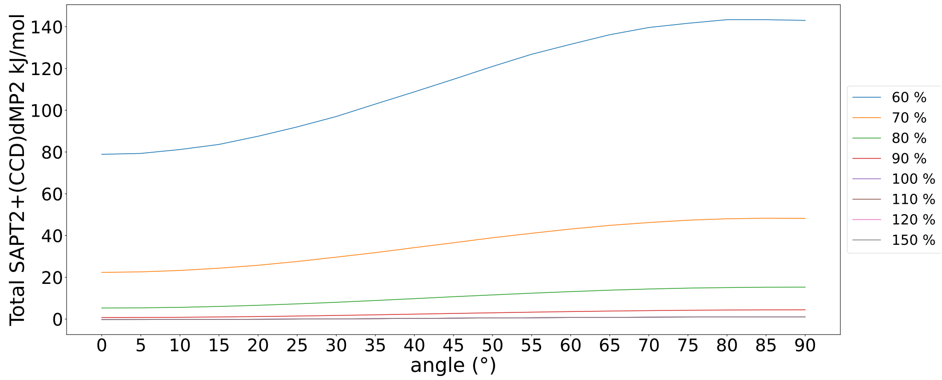

Figure S21: HBr-He, total int,

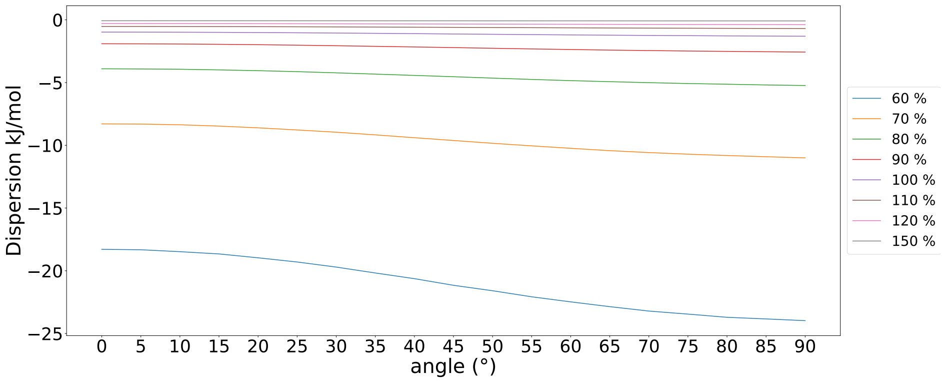

Figure S22: HCl-He, dispersion

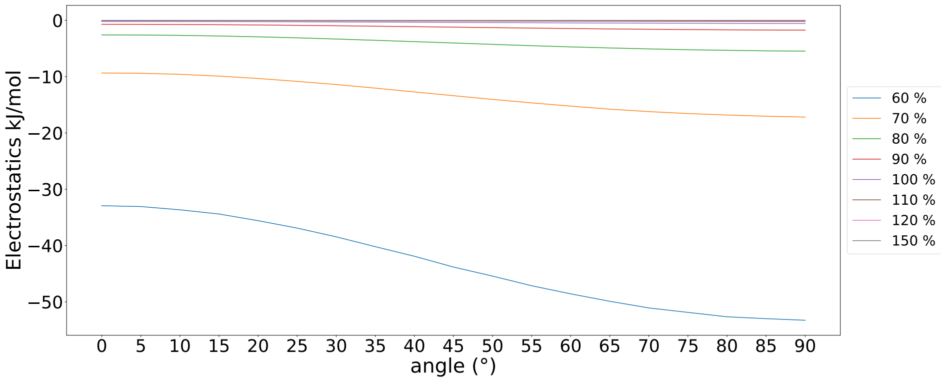

Figure S23: HCl-He, electrostatics

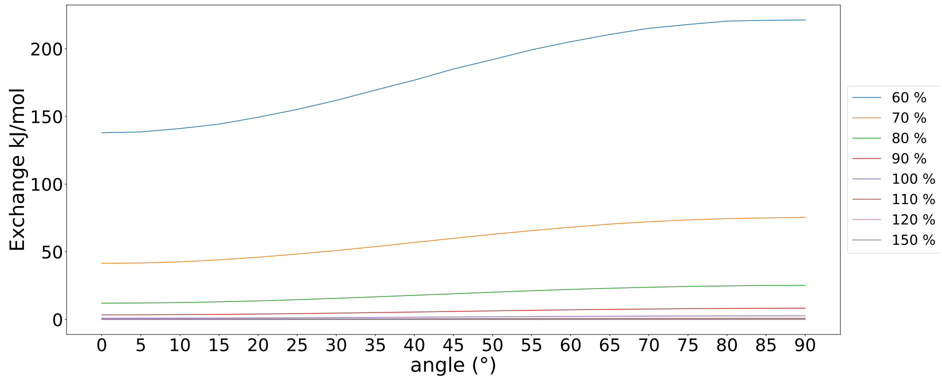

Figure S24: HCl-He, exchange

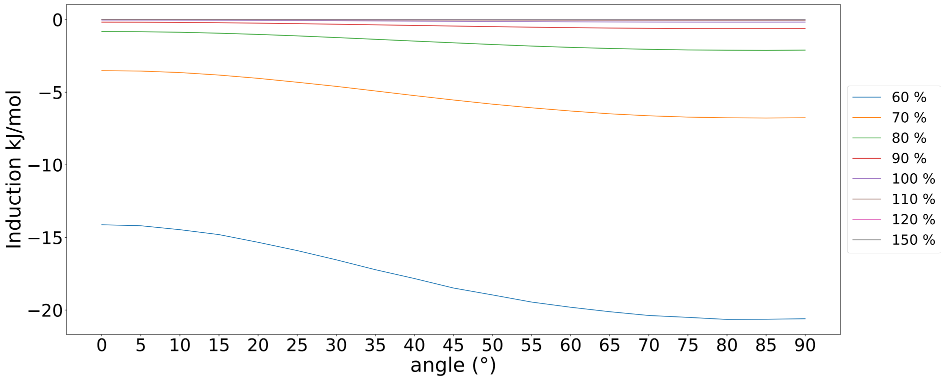

Figure S25: HCl-He, induction

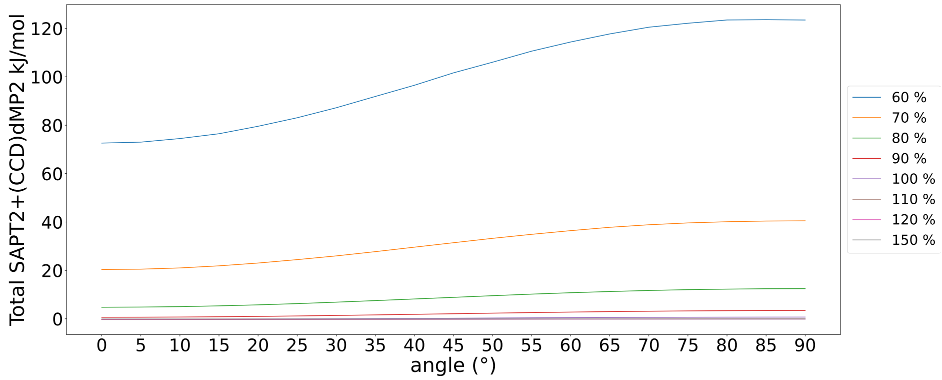

Figure S26: HCl-He, total int,

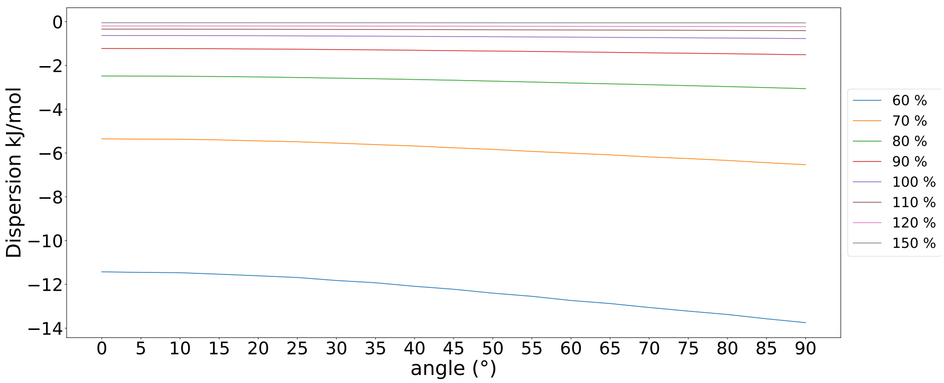

Figure S27: HF-He, dispersion

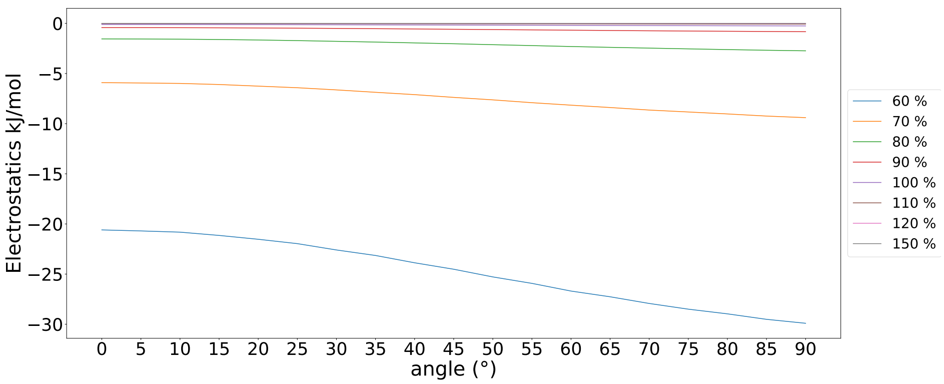

Figure S28: HF-He, electrostatics

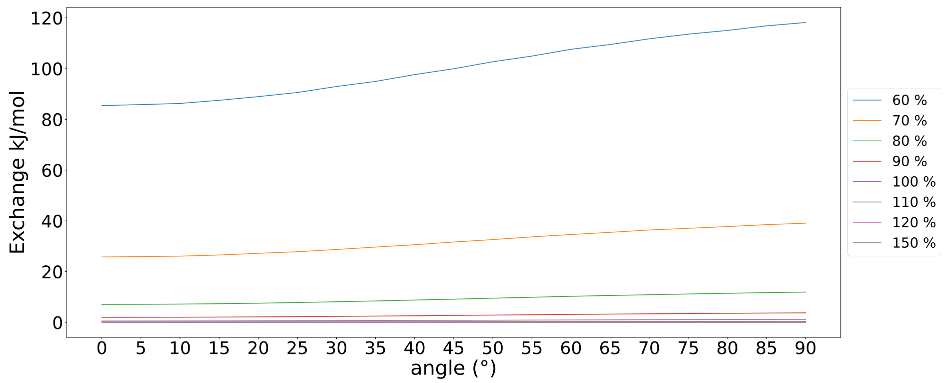

Figure S29: HF-He, exchange

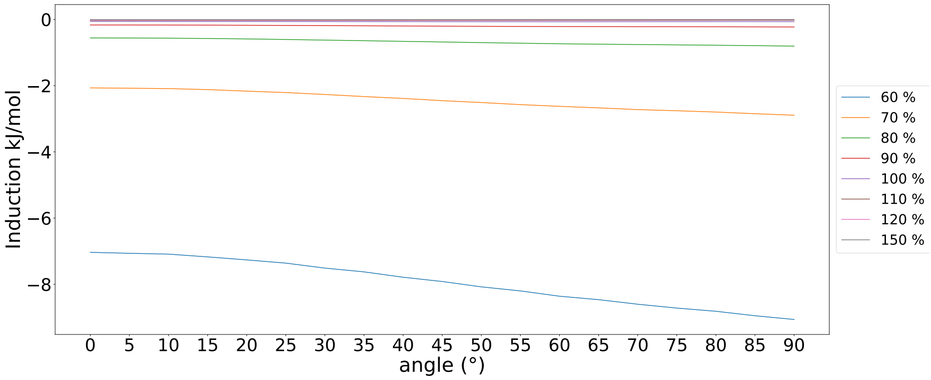

Figure S30: HF-He, induction

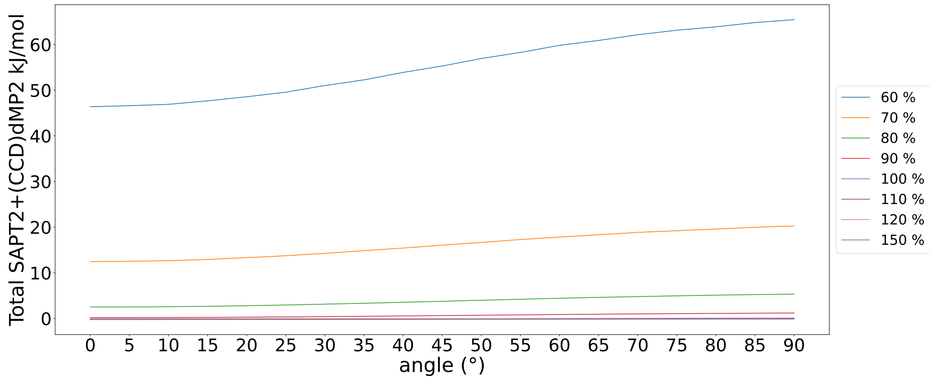

Figure S31: HF-He, total int,
