# Supplementary material for: Quantification of Anisotropy in Exchange and Dispersion Interactions: A Simple Model for Physics-Based Force Fields
Source: J Phys Chem Lett. 2024 Sep 24;15(39):9974–8. doi: 10.1021/acs.jpclett.4c02034 (PMC11457221; doi:10.1021/acs.jpclett.4c02034)
Supplement: Supplementary file 2 — jz4c02034_si_002.pdf [file jz4c02034_si_002.pdf]

jz-2024-02034m.R1

Name: Peer Review Information for "Quantification of Anisotropy in Exchange and Dispersion Interactions: A Simple Model for Physics-Based Force Fields"

First Round of Reviewer Comments

Reviewer: 1

Comments to the Author

Presented is a detailed analysis of the interactions between water or halides with Helium using SAPT ab initio calculations. Results show the need for asymmetric models in the context of empirical force fields to more accurately treat the angle dependence of intermolecular interactions in these simple systems which are associated with presence of lone pairs and sigma holes, an observation that is well known. Interestingly, the study points out the presence of sigma holes in water and suggests including them in force field models. This observation is interesting making the article of wide interest consistent with the readership of JPCL. However, a number of issues that need to be considered by the authors follow.

1) More complex functions in force fields will always improve the treatment of intermolecular interactions. Thus, the improved reproduction of the SAPT data may be considered somewhat trivial. What is most relevant is how the presence of the anistropic models actually impacts the interaction energies between the molecules and He in the context of the water dimer or water-halide interactions. Simply, given that the water dimer interaction energy is approximately 20 kJ/mol,

how much will improved treatment of the lone pairs and sigma holes impact that interaction. Such analysis would include details of the interactions as a function of orientation (similar to the SAPT analysis) but based on the total interaction energy. Are the differences large enough to impact the behavior of molecular liquids, which are representative of the types of systems studied using empirical force fields. Showing this would bring out the importance of the reported observations.

2) Widely used force fields sacrifice the level of agreement with, for example, SAPT data for computational efficiency. This issue should be noted in more details by the authors and estimates of the additional computational demands associated with the suggeted increases in the complexity of the energy functions would be helpful.

3) The importance of the lone pairs being close to perpendicular to the HOH plane of water has been noted and applied in the context of the Drude force field for a water model and, similiarly, for sulfur containing model compounds (JCP 138, 034508, 2013, JCC 31: 2330–2341, 2010).

4) A general presentation issue is the equations and associated tables in the main text. While both of these are important it may be better to place them in the SI as there is minimal information readily accessible to the reader in a table of parameters that clarify the nature of the intermolecular interactions and the role that the sigma holes and lone pairs play in those interactions. This is related to point 1 above.

Reviewer: 2

Comments to the Author

The article by Krız et al. reports a SAPT based study in which the author quantify the anisotropy of exchange and dispersion energy for hydrogen halides and water molecules.

Interestingly, the authors show that this anisotropy can be modeled in force fields via an angle-dependent potential or via a virtual site.

Finally, they suggest that water cannot be neglected when modeling water hydrogen bonds involving and halogen bonding. This interaction can be therefore important also for drug-design proposes

The results reported in the paper are interesting and suitable for the general audience of JPCL.

However, I think that the article would be more impactful if the authors try to apply this approach in more realistic examples.

More importantly, the inclusion of this interaction in the force filed will come at a larger computational cost. To this end it would be important try to quantify how the performance would be with this correction.

In addition, the article would benefit from a better comparison with current available force fields and an introduction that better places the results of the paper in more general content.

Author's Response to Peer Review Comments:

Reviewer(s)' Comments to Author:

Reviewer: 1

Recommendation: This paper is probably publishable, but major revision is needed; I do not need to see future revisions.

Comments:

Presented is a detailed analysis of the interactions between water or halides with Helium using SAPT ab initio calculations. Results show the need for asymmetric models in the context of empirical force fields to more accurately treat the angle dependence of intermolecular interactions in these simple systems which are associated with presence of lone pairs and sigma holes, an observation that is well known. Interestingly, the study points out the presence of sigma holes in water and suggests including them in force field models. This observation is interesting making the article of wide interest consistent with the readership of JPCL. However, a number of issues that need to be considered by the authors follow.

1) More complex functions in force fields will always improve the treatment of intermolecular interactions. Thus, the improved reproduction of the SAPT data may be considered somewhat trivial. What is most relevant is how the presence of the anistropic models actually impacts the interaction energies between the molecules and He in the context of the water dimer or water-halide interactions. Simply, given that the water dimer interaction energy is approximately 20 kJ/mol, how much will improved treatment of the lone pairs and sigma holes impact that interaction. Such analysis would include details of the interactions as a function of orientation (similar to the SAPT analysis) but based on the total interaction energy. Are the differences large enough to impact the behavior of molecular liquids, which are representative of the types of systems studied using empirical force fields. Showing this would bring out the importance of the reported observations.

RESPONSE:

The quality of total interaction energy with respect to SAPT depends on functions applied for the other terms, while we attempted to stay general in our analysis.

We added a new figure 3, showing the incremental decrease in RMSE when adding virtual sites on the lone-pairs or sigma-holes, or both. The exchange RMSE is reduced from approximately 2.5 to 0.5, which is highly significant on the scale of the water-water interaction energy of 20 kJ/mol.

These changes, although not exceedingly large, may well tip the balance between the minimum and close to minimum energy conformations, affecting the optimum geometry of water dimer, and subsequently liquid simulations.

The effect and its extent we are set on investigating further but that falls outside the scope of this paper.

2) Widely used force fields sacrifice the level of agreement with, for example, SAPT data for computational efficiency. This issue should be noted in more details by the authors and estimates of the additional computational demands associated with the suggeted increases in the complexity of the energy functions would be helpful.

RESPONSE:

The computational demands depend on implementation and possible optimizations. For instance, perhaps having joint virtual site for electrostatics and van der Waals does not produce significant error, compared to having them separately. Or perhaps the anisotrophy in real force field would be sufficienty well described adding only one set of virtual particles, omitting the sigma-holes or vice versa. This remains to be investigated but we have added a paragraph in the discussion dedicated to computational costs.

3) The importance of the lone pairs being close to perpendicular to the HOH plane of water has been noted and applied in the context of the Drude force field for a water model and, similiarly, for sulfur containing model compounds (JCP 138, 034508, 2013, JCC 31: 2330–2341, 2010).

RESPONSE:

This article is now properly referenced.

4) A general presentation issue is the equations and associated tables in the main text. While both of these are important it may be better to place them in the SI as there is minimal information readily accessible to the reader in a table of parameters that clarify the nature of the intermolecular interactions and the role that the sigma holes and lone pairs play in those interactions. This is related to point 1 above.

RESPONSE:

The tables have been moved to SI.

Reviewer: 2

Recommendation: This paper may be publishable, but major revision is needed; I would like to be invited to review any future revision.

Comments:

The article by Krız et al. reports a SAPT based study in which the author quantify the anisotropy of exchange and dispersion energy for hydrogen halides and water molecules.

Interestingly, the authors show that this anisotropy can be modeled in force fields via an angle-dependent potential or via a virtual site.

Finally, they suggest that water cannot be neglected when modeling water hydrogen bonds involving and halogen bonding. This interaction can be therefore important also for drug-design proposes

The results reported in the paper are interesting and suitable for the general audience of JPCL.

However, I think that the article would be more impactful if the authors try to apply this approach in more realistic examples.

RESPONSE:

The supporting information contain scans of water and hydrogen-fluoride as a probe. But as that brought no/little extra content, we decided not to discuss it. Now it is mentioned in the text, if a reader would be interested.

We now also include the results of force field training on the same dataset used for empirical fit (using extra virtual sites in the new Figure 3).

Even more realistic exampes will be probably a subject of matter for another publiation.

More importantly, the inclusion of this interaction in the force filed will come at a larger computational cost. To this end it would be important try to quantify how the performance would be with this correction.

In addition, the article would benefit from a better comparison with current available force fields and an introduction that better places the results of the paper in more general content.

RESPONSE:

We have added a part of disccusion devoted to computational costs.

Several contemporary force fields that also adress the anisotropy have been mentioned in introduction. Broad comparison of/to all commonly used force fields we believe is out of scope of this letter.
